# Parental Role Changes in Romanian Transnational Families: Consequences of Migration

**DOI:** 10.3390/ijerph182412960

**Published:** 2021-12-08

**Authors:** Aniela Matei, Elen-Silvana Bobârnat

**Affiliations:** National Scientific Research Institute for Labour and Social Protection 1, 010643 Bucharest, Romania; silvana.bobarnat@incsmps.ro

**Keywords:** parental roles, migration, transnational family, family functions

## Abstract

Even if there are consistent studies on the issue of transnational families, research is still needed to address the parental role changes in these families. The aim of this article was to identify the main changes in the parental roles of Romanian transnational families as a result of the parents’ labor migration. We used interviewing as the research method and directed content analysis to analyze the data. Purposive sampling was conducted in order to identify the interviewees. The results captured important role changes regarding the income provider role of the parent, but especially the role of emotional support provider that the parent should take on for the children. These findings highlight the need to develop specific measures to address the possible negative effects that affect these transnational families.

## 1. Introduction

In the specialized literature [1], transnational families are defined as those families in which family members live separately most of the time but who share a feeling of collective welfare and unity, namely, familyhood, even across national borders. Transnational families are the effect of transnational migration, which is characterized by the fact that migrant people do not sever ties with their country of origin in order to integrate into the culture of the host country but maintain and develop permanent social relations beyond national borders, with family members and others, and identify, through their spatial mobility, with several countries and communities [2].

The term “transnationalism”, which has led to an important debate in the recent literature on migration, was first used by anthropologists Shiller et al. [3] and referred to the social fields that were built by emigrants. The literature in this field [1,4,5] indicates that the use of the transnational approach in the conceptualization of the family, but also in the research methodologies that are used to study transnational families, is recent. Numerous researchers have incorporated transnational studies into family studies by analyzing the consequences of a transnational lifestyle for children with migrant parents [5,6].

Even if there are consistent studies on the issue of transnational families, research is still needed to address the parental role changes in transnational families, with a focus on parental absence in the process of migration, in order to determine the scale for policy interventions in the context of changes in the parental role [7].

At the end of 2019, data that was recorded by the National Authority for the Protection of the Rights of the Child and Adoption in Romania indicated that the phenomenon of transnational families was more pronounced in the case of families where one parent went to work abroad out of the total families that were abroad. In the period 2008–2019, there was an upward trend in the number of single-parent families working abroad, from a total of 37,356 families in 2008 to a total of 48,427 in 2019, which is an approximately 30% increase in that period. Despite the existence of legal provisions to quantify the phenomenon, the official data does not capture its magnitude, especially in the case of families where the parents are not the only supporters of the family; thus, there is a need for empirical research.

The scope of the present research was to identify the effects that performing parental roles had on the children that were left in the charge of other family members in their origin country. We aimed to identify the way in which the income provider role and the emotional support provider role were performed in transnational families and the effects that performing these roles had on the children’s emotional experience and behavior. The research objectives were to identify the children’s emotional and behavioral changes (as observed and described by the child carer) after the parent(s) migration, to describe the ways in which the migrating parents performed in their parental roles after migrating (income and emotional support provider), to verify whether there was any relationship between the emotional and behavioral states of the children and the performance of the income and emotional support provider roles and to identify other factors that influenced the emotional and behavioral states of the children.

## 2. Migration for Work and Parental Role Changes

The literature that has developed over time indicates that the decision regarding migration is made within the family [8]. The factors that were analyzed in the emigration decision can be grouped into the following several categories: economic factors, such as the salary level, income level, daily living costs, unemployment rate (or, in other words, job availability), quality of public services that the state provides them, availability and generosity of the social assistance and social insurance system, and distribution of income; factors related to human capital, such as the age of the person intending to emigrate and their level of education [9]; cultural factors, such as the language spoken in the host country and the cultural norms that exist in that country; social factors, such as the existence of a network of extended family members in the host country [10] or the existence of a community from the same cultural space as the person intending to emigrate [11]; historical factors, which consist of economic relations or relations between the host country and the country of origin [12]; and geographical factors, such as the actual distance between the two countries [12].

The absence of parents affects the exercise of the roles and responsibilities of the family members that remain in the country [13,14,15]. Both the material and intangible resources of the family are affected because families are not only providers of material resources but also social agents with a role that involves providing emotional support. In transnational families, social costs, such as dropping out of school, deviant behavior, or even delinquency, require social policies that are designed to come into play in order to support the family members left at home. Studies on reducing the negative effects of migration [13,16,17,18,19] have revealed the need to develop types of policies/measures/actions at all levels of intervention in order to include the social groups that are affected. The decision to emigrate may have negative consequences on those who remain in the country, with the parents not always being aware of this.

Research in this field [4,19] has shown that the emotional problems of children that are left by their parents in their country of origin are accentuated when their parents start a new life with a new family in the country of migration. The separation between parents and children due to migration also generates social costs, with the role of the child’s caregiver being taken over by another person or by state institutions. Moreover, the same research indicates that migrant families experience reconfigurations and changes in the roles that are played by family members. The children end up taking over the roles of the departed parent, taking on tasks that were normally performed by parents. According to the cited research, this generates a loss of interest in school until the child drops out of school.

The educational activity is directly affected by the parent’s migration in most cases, especially when the mother is the one who migrates [13]. The mother has a central role in the care and education of a child, especially at a young age. Research in the field [20] indicates that children’s educational outcomes may differ depending on who the migrant parent is and the parent in whose care the child remains.

In addition to the opportunities that are created by transnationality, this phenomenon also raises questions about how the family continues to fulfill its functions. As indicated by international scientific studies [21,22,23], the problems of transnational families and the exercise of the function of childcare are widely researched phenomena. However, there are studies [24] that argue that the obligations of transnational families toward children must be seen in the European context by respecting their right to work and social protection.

In Romania, the specialized literature [24,25,26,27] indicates an increase in the phenomenon of Romanian citizens migrating outside the country in recent years, which has affected the structure and functionality of the family in general. When children are left behind in their country of origin, emigration becomes a destabilizing factor for the family, even if there are several economic benefits. Thus, for families, and especially for those with children, migration brings changes in terms of family functions, including the status and roles of family members [28]. Children who remain in their country of origin maybecome a social problem when a person is not designated by the family to take care of them. The social impact can be high, especially in poor communities, where state intervention is needed. Table 1 describes positive and negative outcomes of parent’s migration on Romanian children.

Although children are not always the focus of migration research, they are certainly the focus of transnational family life. Studies that analyzed the condition of childhood in the migration process tended to focus on the second generation of migrants and, as such, on the process of assimilation in the host country, language learning, and school integration [32]. Studies on transnationalism [15,32], even those focusing on the transnational family and the effects of adult migration on children, were conducted from the perspective of the adult experience by considering only their point of view. In contrast, studies that analyzed the role of children in the migration process of their parents by considering their point of view as social actors in transnational families are very rare [15,33].

## 3. Materials and Methods

### 3.1. Data Source and Research Questions

The data source that was used in this article was a collection of qualitative interviews that were carried out within the project “Effects of migration experiences on families, children, and communities left behind: methods of assessment and strategies for mitigating the risk of social exclusion”. A total of 24 interviews were conducted with parents/grandparents from families who had children up to 17 years old and who had a parent (or both parents) who went to work outside Romania (12 interviews in the Northeast Region of Romania and 12 interviews in the Southeast Region of Romania). The selection of development regions was made based on the analysis of indicators that were related to poverty and temporary and permanent migration, and we selected the two regions of Romania that were most affected by poverty.

Through the present research, we aimed to answer the following four research questions: RQ1—What emotional and behavioral changes were identified in children by the child carer after the children’s parent(s) migrated? RQ2—How did the migrating parents perform in their parental roles of income and emotional support provider after their migration? RQ3—Were the emotional and behavioral states of the children related to the performance of the income and emotional support provider roles, and how are they related? RQ4—What other factors influenced the emotional and behavioral states of the children (other than the performance of the income and emotional support provider roles)?

### 3.2. Method and Characteristics of the Participants

In order to identify the changes in the parental roles in the context of labor migration, a semi-structured interview guide that addressed the following topics of discussion was designed and used: the role of the socializing agent, the role of the emotional support provider, the role of the income provider, family solidarity, and strategies for maintaining family solidarity. The use of qualitative methods in migration research is recommended in order to address topics of interest for contemporary migration; social processes related to migration; and the relationship between theory, research design, and practice [34].

The sample size took into account the aim of the research, the sample specificity, and the analysis strategy [35,36]. The purposive sampling technique was used; this is a specific sampling technique that is used in qualitative research since it is suitable for choosing respondents for qualitative studies that use interviews as a data collection tool [37].

The following criteria were taken into account in order to select the participants for the semi-structured interviews:

- The quality of the respondent’s relationship to the child: parent/grandparent to ensure there was a balanced distribution from both categories;
- Migration experience of the parent/parents for work abroad: to have an experience of at least 6 months;
- The districts included in the study: to include at least two districts in the research;
- Residence environment: urban/rural in order to reflect the structure of the phenomenon, mainly from rural and small towns;
- Age of the children: to cover as many cases as possible from the earliest ages up to the maximum age limit of 17 years.

Potential respondents were identified on a network basis using well-known teachers and community facilitators (postman or other community leaders) who were able to provide potential respondents that meet the required criteria. The county coordinator drew up a list of potential respondents who were contacted later in order to confirm their fulfillment of the criteria and to obtain their agreement to participate in the study.

The semi-structured interviews were conducted face-to-face at the home of the respondents between 6 July and 13 July 2021 and they were audio recorded while respecting GDPR rules. The data collection process was carried out after obtaining the acceptance of the interviewed participants, with the data collected while respecting the principles of anonymity and confidentiality. Table 2 describes the characteristics of the sample.

### 3.3. Data Analysis

We used a directed content analysis approach [38] and the following two methods of *describing* the data: *coding* the data and creating *matrixes* [39]. We first employed a description of the data by organizing it by codes. The initial codes were developed based on the existing literature on parental roles and the effects of parents’ migration on children, and then we created new codes that emerged from the collected data. The coding was made based on specific rules established by the authors, rules that are available in Appendix A.1. Data Registration Rules. In the process of analyzing the data, the authors identified when data saturation was achieved [40]. The synthetic description of the data is available in Appendix A.2. Concept Map. Further, we generated matrixes with codes in order to make comparisons and note relations between variables. The software used was NVivo 12 Plus, and the functions that were run were Nodes, Project Map, and Matrix Coding.

## 4. Results

The participants in the semi-structured interviews were either the parents or the grandparents of the children from transnational families. In these families, one or both parents migrated abroad for economic purposes.

### 4.1. Changes in Emotional and Behavioral States of the Children after the Migration

The child carers that were interviewed either did not observe any changes in the children left in their charge or observed changes that could be categorized as emotional changes and behavioral changes. We further analyzed in detail the children’s emotional and behavioral states after the parent’s migration, answering the first research question.

When looking at the emotional experiences of the children after the parent(s) migration, we identified the following three types of cases: (1) some of the children did not experience any emotional changes after the migration of their mother/father abroad, (2) others experienced pleasant emotional experiences, but the vast majority of the children (3) dealt with unpleasant emotional states.

When further looking at the cases that reported unpleasant emotional states of the children, the following two types of cases were identified: (1) Short-term and low-intensity unpleasant emotional experiences that lasted minutes, hours, or days and that manifested infrequently, especially after the departure of the parent(s), after talking on the phone with the migrating parents, or in moments that the children would usually spend with their parents; we also include here those children who had high-intensity feelings but overcame them before the interview. (2) Long-term and high-intensity unpleasant emotions that manifested for months or that became permanent and that the children were still dealing with at the time of the interview.

When looking at the behavioral states of the children with migrating parent(s), the following three general behaviors emerged: isolation from family members and colleagues, school absenteeism, and other risky behaviors (self-harming behaviors, home-absenteeism). Of course, not all children manifested these behaviors; therefore, for each behavior, we identified the following two types of cases: (1) the cases in which the children manifested the behavior, and (2) the cases in which the children did not manifest it.

Based on these emotional and behavioral states, we identified four types of cases. Those four emotional–behavioral types contributed to making relevant comparisons and identifying risk factors for the children.

▪ Emotional–behavioral type one: the most vulnerable type consisted of cases in which the children manifested all three risky behaviors—isolation from family members and colleagues, school absenteeism, and other risky behaviors—and had long-term high-intensity unpleasant feelings.
▪ Emotional–behavioral type two: this emotionally vulnerable type consisted of cases in which the children experienced long-term high-intensity unpleasant emotions and also some degree of isolation, but they did not engage in school absenteeism or other risky behaviors.
▪ Emotional–behavioral type three: this “at risk of dropping out of school” type consisted of cases in which the children started missing school or had long periods of school absenteeism without isolating him/herself from others, without having other risky behaviors, and without having long-term high-intensity unpleasant emotional states.
▪ Emotional–behavioral type four: the most resilient type consisted of cases in which the children did not engage in any of the behaviors that were previously mentioned, regardless of the feelings they experienced after their parent(s) migrated (because of the criteria consisting of the children not experiencing isolation from family members and colleagues, school absenteeism, and other risky behaviors, the children with high-intensity long-term unpleasant feelings were automatically excluded from this category and included in one of the first two categories because those who, at the time of the interview, manifested emotionally disruptive states were also experiencing at least one of the mentioned behaviors).

When analyzing the four emotional–behavioral types by age and number of children, it emerged that the most vulnerable and the emotionally vulnerable types included families with a small number of children, all over 14 years of age; the “at risk of dropping out of school” type included families with a large number of children (5–6 children), with ages both under and above 14 years old; and the most resilient type was heterogeneous regarding the number of children and their age, though it generally included families with a small number of children, but also some with a larger number of children (5), and generally children younger than 14 years old.

At this point in the research, the age of the child could be considered a risk factor for long-term high-intensity unpleasant emotions and also some degree of isolation, while the number of children (between five and six) could be a risk factor for school absenteeism, which partially answered the fourth research question (RQ4—What other factors influenced the emotional and behavioral states of the children (other than the performance of the income and emotional support provider role)?). Table 3 describes the emotional–behavioral types by the number of children and age.

In the next few sections, we provide the results of the performance analysis of the parents and the child carers as the income and emotional support provider roles, and their effects on the children’s well-being.

### 4.2. The Income Provider Role Performance

In this section, we provide the results of the performance analysis of the migrating parent(s) in their role as income provider for the children, and also the effects of this role’s performance on the children’s emotional and behavioral states, partially answering the second and the third research questions.

Prior to migration, some of the families faced extreme material deprivation (lack of food, for example), while others were unable to buy a larger house or send the children to university due to their financial burdens. In all the cases included in the research, the poor performance of the role of income provider motivated the parents to decide that one or both of them should migrate.

After migration, the majority of the migrating parents became (1) *constant income providers* for their families, but in some of the cases, the parents who left to work abroad (2) *disengaged from their role of income provider*. After migration, some of the families faced (1) *extreme material deprivation* (lack of food, clothes, and heating in the winter), while (2) the others *improved their material status*, financially covering not only day-to-day expenses but also enhancing their living conditions and children’s participation in non-formal education activities.

Regarding *how the migrated parents performed in their parental role of income provider after migration*, the following four types of cases could be distinguished: (1) the cases in which the parents were constant income providers and were able to fulfill the basic needs of their families (income provider performance type one); (2) the cases in which the parents were constant income providers but the basic expenses of the families were not fulfilled, and thus, the children faced extreme material deprivation (income provider performance type two); (3) the cases in which the parent disengaged from their role of income provider and the children faced material deprivation (income provider performance type three); and (4) the cases in which the parent disengaged from their role of income provider but the basic expenses of the families were covered by the child carer (income provider performance type four).

When we look at the emotional–behavioral types and income provider performance types, we found that *the most vulnerable* type was also characterized by parents who disengaged from their role as income provider and children who faced material deprivation (income provider performance type three), *the emotionally vulnerable* type was characterized by parents who disengaged from their role as income provider but the children did not face extreme material deprivation due to the role being fulfilled by other members of the family (income provider performance type four), *the “at risk of dropping out of school*” sample was characterized either by parents who were constant income providers but are unable to cover the basic expenses of the families or by parents who were constant income providers and the basic needs of the families were fulfilled (income provider performance types one and two), and *the most resilient* sample was characterized by parents who were constant income providers and the families had their day-to-day expenses for basic needs fulfilled (income provider performance type one).

When we looked further at the “*at risk of dropping out of school*” type that had *parents who were constant income providers* and *did not face severe material deprivation* (emotional–behavioral type three and income provider performance type one), we found that this specific category was characterized by having a large number of children (six children), where one or two of the children aged over 14 years are already working and bringing money home, and thereby contributing to the fulfillment of the basic needs of the family.

“*He goes to work. They [the children] also bring money home, the father from one side*…”.

(Mother, NE region, six children)

The “*at risk of dropping out of school*” type with parents who were constant income providers but were unable to cover the basic expenses of the families was also characterized by a large number of children (five children), but all of them were under 14 years.

“*The boy, the girl… They feel the need to leave school to go to work… Not all of them… I brought them back from a sheepfold [where they were working]… and I told [to the shepherd] not to receive them [at the sheepfold to work] any more. They feel the need to work. When I needed help, he ploughed in the garden, weeded. <Grandma, I’m going to work.> < You are not working, you are 10 years old*>”.

(Grandmother, NE region, five children)

At this stage of the analysis, it emerged that the unfulfillment of the family basic needs (food, clothes, and heating in winter) (types two and three of the income provider role performance) was a possible factor for dropping out of school (one of the risky behaviors of the children that were identified by child carers). Another risk factor for dropping out of school was a large number of children, a factor that was also identified in the previous section, which partially answered the fourth research question (RQ4—*What other factors influence the emotional and behavioral states of the children*?).

However, there were also families with a large number of children (five children) that did not disengage from school participation, and there were also families with a small number of children that missed school, with other factors that were also important in school absenteeism that needed further analysis.

If a family fails to “successfully discharge” [38] the financial responsibilities that are incumbent on it, society, through its other institutions, intervenes (e.g., the church, the school, non-governmental organizations, Child Protection Service), primarily by supplementing the family income or providing material aid: social assistance, material aid in the form of supplies or electronic devices (tablets) received through schools, food received through school canteens, and material aid received from various NGOs or the priest of the community. Table 4 describes emotional–behavioral types by income provider performance type.

### 4.3. The Emotional Support Provider Role Performance

The emotional closeness of the children to the child carer prior to and after migration, the emotional closeness of the children to the migrated parent(s) prior to their migration, and how well the migrated parent(s) performed in their role as a provider of emotional support after their migration, including emotional closeness, frequency of visits, and frequency of interactions through ICT, were all analyzed and were related to the children’s emotional and behavioral states. In this section, we aimed to partially answer the second, third, and fourth research questions.

When we looked at *the emotional closeness of the children to the child carer* (either being one of the parents or the grandparents), we distinguished the following three types of cases: (1) the children were close to the child carer prior to migration and continued being close (child–carer emotional closeness type one—good/good), (2) the relationship between the children and the child carer was a distant one, but after the parent(s) migrated, the two became emotionally closer (child–carer emotional closeness type two—poor/good), and (3) the relationship between the children and the child carer was a distant one, and they continued being distant even after the migration (child–carer emotional closeness type three—poor/poor).

When relating the emotional closeness of the children to the child carer using the emotional–behavioral types, the following results emerged (described in Table 5):

- *The most vulnerable* type (high-intensity long-term unpleasant feelings, school absenteeism, isolation, and other risky behaviors) was characterized by a poor emotional closeness between the children and the child carer prior to but also after migration;
- *The emotionally vulnerable* type (high-intensity long-term unpleasant feelings and some degree of isolation, but no other risky behaviors or school absenteeism) was characterized by a good emotional closeness between the children and the child carer prior to and after migration (suggesting that the good emotional relationship with the child carer could be a buffer between different risk factors and the children);
- *The “at risk of dropping out of school”* type (school absenteeism) was characterized by a good emotional closeness between the children and the child carer prior to and after the migration (suggesting other factors of school drop-out risk for this category of children); and
- *The most resilient* type was characterized by a good emotional closeness between the children and the child carer prior to and after migration but also included cases in which the relationship was poor prior to migration and it improved over time after the migration, which emphasized the importance of the relationship between the two of them after the migration. Table 5 describes emotional–behavioral types by the emotional closeness of the child to the child carer.

**Table 5:** Emotional–behavioral types by the emotional closeness of the child to the child carer.

Emotional–Behavioral Types	The Emotional Closeness of the Child to the Child Carer	Status of the Child Carer
Type 1 (the most vulnerable)	Poor emotional closeness between the children and the children carer prior to but also after migration.	Grandparent
Type 2 (the emotionally vulnerable)	Good emotional closeness between the children and the children carer prior to and after migration.	Parent
Type 3 (at risk of dropping out of school)	Good emotional closeness between the children and the children carer prior to and after migration.	Grandparent, parent
Type 4 (the most resilient)	Good emotional closeness between the children and the children carer prior to and after migration, but also included cases in which the relationship was poor prior to the migration and it improved after migration.	Grandparent, parent

The analysis of these types of emotional closeness to the child together with the child carer status (parent/grandparent) indicated that all child carers that were parents had a child carer emotional closeness type one—good/good emotional closeness with the children, and that the child carers that were grandparents may have had all three types of emotional closeness to the children (good/good, poor/good, poor/poor), suggesting that there were smaller emotional risks for the children when the child carers were the parents, with the mother being the most significant one for the child (further analyses are needed regarding the gender of the child carer and whether the child carer’s gender was more important than the child carer’s status).

In relation to the migrating parent(s), we analyzed their emotional closeness prior to their migration, and we related this variable with the emotional–behavioral types, but the results were more relevant when we analyzed *the emotional closeness between the child and the migrating parent(s)* prior to migration in relation to *the emotional experience of the child after migration*.

The children who experienced a poor emotional closeness with the migrating parent also experienced pleasant emotional states due to the parent migration;

*Not much [were the children attached to the father before the migration] … he was only swearing … No one answers [when the father calls at the phone]… He put his hands in his pockets, and he started swearing, even when he sat at the table. It’s better without him*.

(Mother, SE region, six children)

The children who were emotionally close to their migrating parents either had no changes in their emotional state or experienced unpleasant feelings. Of those who experienced unpleasant feelingssome had (a) short-term low-intensity feelings, including those who had had high-intensity feelings but had overcome them at the time of the interview, while others faced (b) long-term high-intensity emotional states.

*[The child] misses them [the parents]. <I care about you, but I miss my mother and my father that I can’t take it anymore> [says the child]*.

(Grandmother, NE region, one child)

*I think she suffers from the absence of her mother, less of her father … but first of all of her mother. That is why she tattoos signs that remind her of her mother or signs that would draw her mother’s attention because she misses her*.

(Grandmother, SE region, one child)

At this point in the analysis, the child–migrated parent(s) emotional closeness *prior to migration* emerged as another possible factor for the child’s emotional experience after the migration (fourth research question).

Table 6 describes emotional experience of the child by emotional closeness to the migrating parent prior to the migration.

We further analyzed *the performance of the role of emotional support provider of the migrated parent(s)* (which included the variables emotional closeness, frequency of visits, and frequency of interactions through ICT), and three levels of performance were distinguished: *good*, *medium,* and *poor*, which related to the emotional–behavioral types.

*The most vulnerable* type (high-intensity long-term unpleasant feelings, school absenteeism, isolation, other risky behaviors) was characterized by *poor* performance of the migrating parents in their role as a provider of emotional support. The ICT-mediated interaction was infrequent, the parents neither came home for years nor invited the child and the child’s carer to visit them abroad, and the child did not communicate important intimate things about their life to their parent(s) (e.g., about their self-destructive behavior). These children faced long-term unpleasant emotional feelings, tended to isolate themselves from family and colleagues, skipped school, and were at risk of self-destructive behaviors.

*Once a week, every 2 weeks … not often* [the child talk on the phone, WhatsApp, Facebook with the parents].

(Grandmother, SE region, one grandson)

*Maybe if we had been invited [to visit the child’s parents abroad] we would have thought going … but in the absence of an invitation …*

(Grandmother, SE region, one grandson)

*C. was a very cheerful child, ready to help all her friends, all her colleagues … sociable. After her parents left, she became an introvert … she cried … she cut her hair short … she tattooed her right hand, her left arm. It is quite difficult to talk to her, I find out a lot of things about her from the teachers… At first I had the impression that she isolated only from me, but I found out from the teacher that she isolated herself from her friends, her colleagues … sometimes I see that she cut her hands … [The niece] says that it is a form through which she wants to see how much she resists pain*.

(Grandmother, SE region, one child)

*I don’t think [the child told the parents about the self-destructive behaviour]… they would have asked me*.

(Grandmother, SE region, one child)

*The emotionally vulnerable* type (high-intensity long-term unpleasant feelings and some degree of isolation but no other risky behaviors and school absenteeism) was also characterized by the *poor* performance of the migrated parents in their role as an emotional support provider. The children experienced long-term emotionally disruptive states and, to a certain degree, they isolated themselves from their family members. However, compared to the most vulnerable, the emotionally vulnerable had a good closeness with their migrated parents, which could attenuate the effects of the migrated parent’s poor performance at providing emotional support to the children.

The “*at risk of dropping out of school*” type (school absenteeism) was characterized by at least the medium performance of the migrating parent(s) in their role as a provider of emotional support.

The *most resilient* were characterized by the good or at least moderate performance of the migrating parents in their role as a provider of emotional support: frequent ICT-mediated interactions; the parent(s) returning home multiple times a year, especially in strongly socially regulated contexts (holidays, birthdays, and graduations); the children visiting their parents abroad; and the intimacy of the discussions between the children and the migrated parent(s).

*On another trip to skiing too … he takes him on trips every year. And I think this year the child will spend the holidays in France with the parents… Yes, they always come home, and they stay 2-3 months … for example, the mother has gone abroad only 1 month ago. … She stayed at home with him for 1 month. There aren’t the kind of parents that forget the child at home*.

(Grandmother, NE region, one grandson)

Table 7 describes emotional–behavioral types by the emotional performance support types.

**Figure 1:**
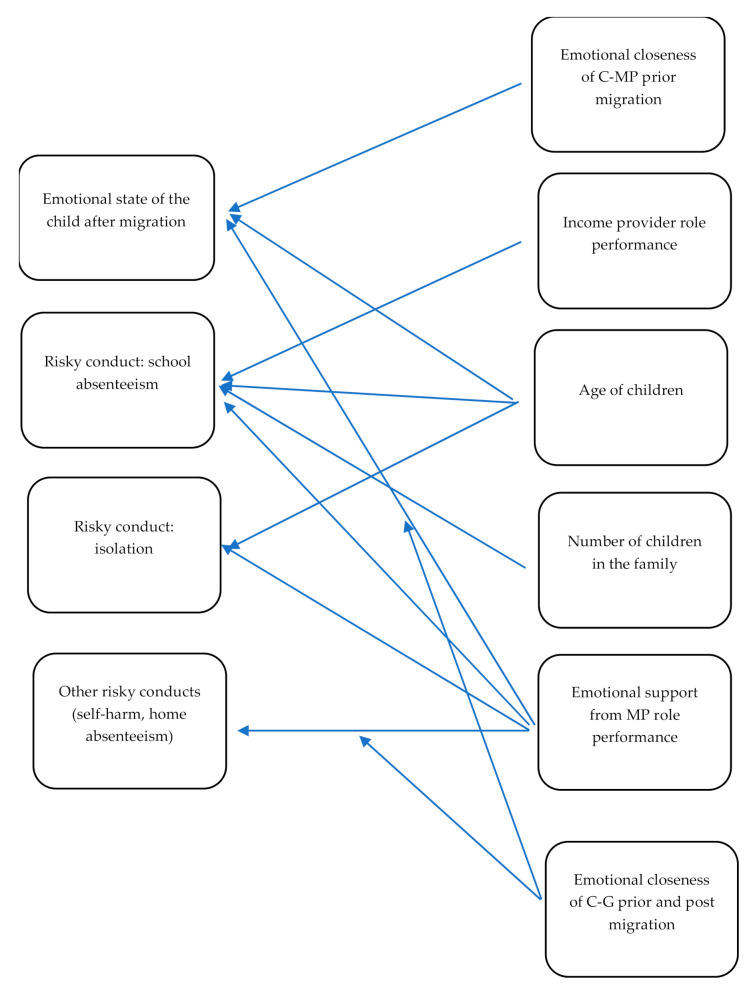
The factors of post-migration child experience.

As a conclusion to this section, a poor emotional closeness between the children and the migrated parent(s) prior to their migration was related to the children’s emotional state in the sense that the migration abroad of parents with whom the children were poorly connected enhanced the children’s emotional well-being; a poor emotional support offered to children by their migrating parent(s) was related to high-intensity long-term unpleasant feelings and the three risky behaviors (school absenteeism, isolation, other risky behaviors), but a good emotional closeness between the child and the child carer acted as a buffer between the poor emotional support offered to the children by their migrating parent(s) and the following two behaviors: school absenteeism and other risky behaviors. In Figure 1, the conclusion of the chapter is synthesized (abbreviations used: MP (migrated parent(s)), C (child), G (guardian/child carer)).

Following this research, some hypotheses that could be tested in quantitative research emerged. They are as follows:

1. The emotional state of the child after the migration of the parent(s) was correlated with the child–migrating parent(s)’ emotional closeness prior to their migration. A poor emotional closeness between the child and the migrating parent(s) enhanced the chances that the child would not experience a disruptive feeling after the parent’s migration.
2. Poor income provider role performance (disengagement from the role of income provider and not fulfilling the basic material needs of the child) enhanced the risk for school absenteeism.
3. The age of the child was correlated with the emotional state of the child and behaviors, such as school absenteeism and isolation.
4. The number of children enhanced the risk of school absenteeism.
5. Emotional support from the migrated parent(s) (emotional closeness, frequency of visits, and frequency of communication) was correlated with the emotional and behavioral states of the child. Poor emotional support enhanced the risk of long-term high-intensity unpleasant emotions, isolation, school absenteeism, and other risky behaviors (e.g., home absenteeism, self-harm).
6. Emotional closeness (especially post-migration relations) to the child’s carer reduced the impact of poor emotional support from the migrating parent(s) on school absenteeism and other risky behaviors.

## 5. Discussion

Due to the migration of one or both parents, transnational families experience changes in the parental role; some of these adjustments improve parental performance, with others being risk factors for the children that are left at home with relatives. The results of our research highlighted that some of the children faced emotional and/or material deprivation (depending on the parental role performance, child carer role performance, structure of the family, etc.), which was related to outcomes later in the children’s lives.

Generally, the economic status of the families—the living conditions, procurement of necessary goods, and covering the expenses for participation in education—improved after migration, which was consistent with previous findings [29,30]. Furthermore, our research highlighted the existence of a category of families in which, even after migration and despite the migrated parents’ effort to constantly provide an income to their family, children lacked appropriate food, clothes, and living conditions, and they were tempted to miss school and go to work in order to contribute to the family income. These families were characterized by a large number of children, and even if they received material support from NGOs, churches, and other family members, they could not surpass their financial burdens. Material deprivation has physical health outcomes, cognitive outcomes, and emotional and behavioral outcomes, as documented by Brooks-Gunn and Duncan [41]. Interventions consisting of food, clothes, and improvement in living conditions could reduce the impact of poverty on children, especially if they are implemented in the early stages of a child’s life. In some families, the prescription of the child’s role as being involved in paid work (an aspect not documented in this study) could be a risk factor for school participation, cases in which specific intervention regarding the appropriate prescription of a child’s role could reduce the risk of school absenteeism.

However, children had dropped out of school even if their family was not materially deprived at the time of the interview. The family’s ability to avoid material deprivation may have been due to the children’s income. In these cases, interventions consisting of reintegrating the children into school or their integration into the formal labor market (benefiting from income protection, safe working conditions, etc.) could reduce the impact of dropping out of school on older children.

Some of the children faced emotional support deprivation due to their parents becoming disengaged from their parental role. The results were consistent with previous findings [29,31] that highlighted the emotional consequences of parents migrating for the children, but our research provided further information on the appropriate performance on parental roles and the child carer role and how these can diminish the risk of negative emotional outcomes. Interventions that consist of counseling could reduce the emotional risk and the associated behaviors. Interventions that target the parents and their prescribed role as a provider of emotional support for their children could change role prescriptions and improve role performance.

Further research could identify other factors of children’s well-being in transnational families (school, peers, etc.), document the obstacles that the child carer and the children face when asking for material support from state institutions or counseling from schools, or could test the hypotheses that were formulated based on the results of this study.

Migration is a critical moment for family solidarity [42,43,44,45,46,47,48,49]. The deterioration of the feeling of family solidarity is amplified by a low frequency of the migrant parent returning to their country and their absence in strongly regulated social moments (holidays, anniversaries, etc.). While for some families, migration has led to a sharp deterioration in family solidarity, other families have maintained or even improved their family solidarity. The adequate fulfillment of parental roles by the migrant parent is an important element of family solidarity.

## 6. Conclusions

The information gathered through semi-structured interviews showed that, in most of the cases, the role of the main income provider of the migrant parent was enhanced. However, there were cases in which the parent failed to fully fulfill their role of income provider such that the children continued to face material deprivation, sometimes even in its severe forms (lack of food, lack of heat in the cold season). These cases were found in families with a high number of children and a lower level of parental education. In the context of the father’s migration and severe material deprivation affecting the family members who remained in the country, the children themselves partially took over the role of income provider, with a simultaneous reduction in school participation, thus outlining the risk of school absenteeism. A third typical case saw the parent gradually disengage from their role of income provider, with a simultaneous total or partial disengagement from their other roles (parental, partner of the conjugal couple, son). In this case, the role of the income earner was taken over by the parent that remained in the country and/or by the grandparents, with its fulfillment being achieved poorly.

The migration of parents abroad had profound implications for the emotional experience of family members, especially children. The negative emotional experience was deepened by the lack of the migrant parent in strongly socially regulated contexts, such as winter holidays, birthdays, celebrations, and sports activities, in which the child’s expectations—compared to other families or pre-migration experience—was for the parent to be present and involved. The absence of the parent was understood by the child as disengagement from parental roles. The disengagement of the migrant parents from the role of emotional support was associated with intense and lasting negative emotional feelings, complemented by isolation, self-destructive behaviors, and absence from school. Negative (internalization, sadness) and lasting emotional feelings were also found in cases where one of the parents disengaged from the role of partner in their marriage but they were not followed by auto-destructive behaviors and school absenteeism.

The free movement of labor in the European space has generated a social problem in Romania: the problem of children whose parents have left to work abroad. The magnitude of the phenomenon of children whose parents have left to work abroad and the need to identify solutions for managing this phenomenon is closely related to understanding this phenomenon, as the empirical research is quite poorly developed. Despite the limitations of this study (geographical coverage was restricted to only two development regions of Romania), the results of this piece of qualitative research provide public policymakers with information to better understand the parental role changes in Romanian transnational families.

## Figures and Tables

**Table 1:** Positive and negative outcomes of parent’s migration on Romanian children.

Positive Outcomes of Migration on Children	Negative Outcomes of Migration on Children
Economic outcomes	
Increase in the economic well-being of the child: financial, living conditions, procurement of goods [29,30] Increase in visits abroad [29,30]	
Educational outcomes	
The completion of secondary and tertiary education [29,30] Better school performance compared to children with non-migrant parents [29,30]	Lack of support regarding school-related issues [29] Deviant behavior at school (small differences compared to children with both parents at home) [29] The migration abroad of a child prior to them finishing secondary education, especially for boys with low-educated parents [29]
Psycho-social outcomes	
A reduction in family conflict (effects identified by the children) [30] Greater degree of freedom for the children [30]	Deterioration of the relationships between the child and the child’s carer parent, especially when the migrating parent is the mother [29] Emotional outcomes (e.g., depression) [29,31] Consumption of alcohol and smoking, especially for children where both parents migrated, or where their mother migrated [29]
Health outcomes	
	More frequent health problems than children with non-migrant parents (other than depression) [31]

**Table 2:** Characteristics of the sample.

Criteria	Statistics
Quality of the respondent	10 of the respondents were grandparents and 14 were parents.
Migration experience	Under 3 years of migration experience/time since the partner/children’s parent(s) went abroad: 4 participants; Between 3 to 5 years of migration experience/time since the partner/children’s parent(s) went abroad: 13 participants; 6 or more years of migration experience/time since the partner/children’s parent(s) went abroad: 6 participants.
Residence	9 interviews were conducted in urban areas and 15 interviews were conducted in rural areas.
The ages of the children whose parents/grandparents were interviewed	In 14 of the 24 interviews, the parent/grandparents cared for more than 1 (one) child/grandchild: - The average age of the child: 10 years; - Percentage of children above the average age: 38.7%; - Percentage of children under the average age: 51.6%.
The countries the parent(s) who went to work abroad migrated to	- Spain: 4 cases; - Italy: 4 cases; - France: 4 cases; - Germany: 4 cases; - England: 3 cases; - Denmark: 1 case; - Norway: 1 case; - Netherlands: 1 case; - Sweden: 1 case; - United States of America: 1 case.

Source: Authors’ development based on the methodological report of the interviews.

**Table 3:** Emotional–behavioral types by the number of children and age.

Emotional–Behavioral Types	Emotional Changes	Behavioral Changes	Number of Children	Ages of Children
Type 1 (the most vulnerable)	Long-term high-intensity unpleasant feelings	Isolation from family members and colleagues, school absenteeism, and other risky behaviors	Small number of children (1 child)	Over 14 years old
Type 2 (the emotionally vulnerable)	Long-term high-intensity unpleasant feelings	Isolation from family members and colleagues	Small number of children (1 child)	Over 14 years old
Type 3 (at risk of dropping out of school)	No changes in emotional status or pleasant emotional status, or short-term, low-intensity unpleasant emotions	School absenteeism	Large number of children (5–6 children)	Ages both under and above 14 years old
Type 4 (the most resilient)	No changes in emotional status or short term, low-intensity unpleasant emotions	No behavior change	Generally, families with a small number of children, but also some with a larger number of children (5 children)	Generally, children under 14 years old, but also some exceptions (children older than 14 years)

**Table 4:** Emotional–behavioral types by income provider performance type.

Emotional–Behavioral Types	Income Provider Performance Types	Description of the Income Provider Performance Types
Type 1 (the most vulnerable)	Type 3	The migrated parent disengaged from their role of income provider and the children faced material deprivation.
Type 2 (the emotionally vulnerable)	Type 4	The migrated parent disengaged from their role of income provider, but the basic expenses of the families were covered by the child carer.
Type 3 (at risk of dropping out of school)	Type 1 * Type 2	The migrated parents were constant income providers and were able to fulfill the basic need of the families (Note: the families have large no. of children and one or two of the children over 14 years were working supplementing family income). The migrated parents were constant income providers but the basic expenses of the families were not fulfilled; therefore, the children faced extreme material deprivation (Note: the families have large no. of children and all children were under 14 years).
Type 4 (the most resilient)	Type 1	The migrated parents were constant income providers and were able to fulfill the basic need of the families.

**Table 6:** Emotional experience of the child by emotional closeness to the migrating parent prior to the migration.

Emotional Experience of the Child after the Migration	Emotional Closeness between the Child and the Migrated Parent(s) Prior to Migration
Pleasant emotional state after the parent(s) migrated	Poor
No changes or unpleasant feelings	Good

**Table 7:** Emotional–behavioral types by the emotional performance support types.

Emotional–Behavioral Types	Emotional Support Performance Types (Migrated Parent(s))	Description of the Emotional Support Performance Types
Type 1 (the most vulnerable)	Poor	The emotional closeness between the child and the migrated parent(s) after their migration was poor, the frequency of communication through ICT was once a week or less, and the frequency of visits was less than once a year.
Type 2 (the emotionally vulnerable)	Poor	The emotional closeness between the child and the migrated parent(s) after their migration was poor, the frequency of communication through ICT was once a week or less, and the frequency of visits was less than once a year.
Type 3 (at risk of dropping out of school)	Medium Good	The emotional closeness between the child and the migrated parent(s) after their migration was poor in some of the cases, but it was good in the other cases, the frequency of communication through ICT was at least once a week, and the frequency of visits was in some cases less than once a year and in the other cases more than once a year.
Type 4 (the most resilient)	Medium Good	

## Data Availability

Data sharing is not applicable to this article.

## Appendix A

### Appendix A.1. Data Registration Rules

**Category**	**Rules of Registration of the Data**
Child’s age	We distinguished between *children younger than 14* and registered the cases in which all the children in the family were younger than 14, *children of 14 years or more* and registered the cases in which all the children of the family were aged 14 years or older, and the third sub-category in which there were *children that were both younger and older than 14*, where we registered all the other cases.
Child’s behaviors—other risky behaviors	Other risky behaviors (home absenteeism, self-harm) were some of the changes in a child’s behavior identified by some of the child carers. We distinguished between *the presence of the other risky behaviors*, where we registered the cases in which the child carer mentioned home absenteeism and/or self-harm and *the absence of other risky behaviors*, where we registered all the other cases.
Child’s behavior—school absenteeism	School absenteeism was one of the behaviors that were mentioned by some of the child carers. We distinguished between *the presence of the school absenteeism behavior*, where we registered the cases in which the child carer mentioned school absenteeism or school abandoning, and *the absence of this behavior,* where we registered all the other cases.
Child’s emotional experience after the migration of the parent	We distinguished between *pleasant* and *unpleasant* emotional experiences of the children after the migration of their parent(s) or *no change*, as described by the child carer. We did not encounter cases where some of the children experienced a pleasant emotional experience and the other children of the same family experienced an unpleasant emotional experience.
Unpleasant emotional experience	When analyzing the data, the following two sub-categories emerged: (1) *Short-term and low-intensity* unpleasant emotional experience, which included registrations of the cases where the child care mentioned that the emotional states lasted for minutes, hours, and days, or were passing emotional experiences; a low intensity of the unpleasant emotional experience was explicitly mentioned; the children had high-intensity feelings but had overcome them at the time of the interview. (2) The children had *long-term and high-intensity* unpleasant emotional experience, which included the cases where the child carer mentioned that the emotional state seemed to be permanent or explicitly emphasized the unpleasant emotional state of the child. We did not encounter cases where some of the children experienced a short-term unpleasant emotional experience and the other children of the same family experienced a long-term unpleasant emotional experience.
Emotional closeness between the child and the guardian prior to the migration	We distinguished between *poor* emotional closeness, where we registered the cases in which the guardian explicitly mentioned not being close to the child or having a conflicting relationship with them, and *good* emotional closeness between the child and the child carer, where we registered all the other cases.
Emotional closeness between the child and the guardian after the migration	We distinguished between *poor* emotional closeness, where we registered the cases in which the guardian explicitly mentioned not being close to the child or having a conflicting relationship with them, and *good* emotional closeness between the child and the child carer, where we registered all the other cases.
Emotional closeness between the child and the migrated parent(s) prior to their migration	We distinguished between *poor* emotional closeness, where we registered the cases in which the child carer explicitly mentioned that the migrated parent(s) was/were not close to the child or had a conflicting relationship with them prior to their migration, and *good* emotional closeness, where we registered all the other cases.
Emotional support role performance of the migrated parent(s)	Consisted of emotional closeness between the child and the migrated parent(s) after their migration, frequency of communication through ICT, and frequency of visits. When the emotional closeness between the child and the migrating parent(s) after their migration was good, the frequency of communication through ICT was multiple times a week, and the frequency of visits was at least once a year, the case was registered as *good emotional support.* When the emotional closeness between the child and the migrating parent(s) after their migration was poor, the frequency of communication through ICT was once a week or less, and the frequency of visits was less than once a year, the case was registered as *poor emotional support.* All the other cases were registered as *moderate emotional support*.
Emotional closeness between the child and the migrated parent(s) after their migration	We distinguished between *poor* emotional closeness, where we registered the cases in which the child carer mentioned a poor emotional connection, the lack of reciprocity, or the existence of a conflict between the child and the migrating parent(s), and *good* emotional closeness between the child and the guardian, where we registered all the other cases.
Frequency of communication through ICT	We distinguished between the following two subcategories: *multiple times a week* and *once a week or less*.
Frequency of visits	We distinguished between the following two subcategories: *at least once a year* and *less than once a year*.
Migrated parent(s) as a constant income provider	We distinguished between the following two subcategories: cases in which *the migrated parent(s) was (were) a constant income provider(s)*, even if the income provided was sometimes in a small amount and did not suffice for the month-to-month expenses, and cases in which *the migrating parent(s) was (were) not a constant income provider(s),* occasionally sending money to the family.
Migration motive	The motive identified in all cases was material support for the family, especially for the children. We also identified the dissolution of the conjugal couple, complementary to material support in one of the cases; therefore, in order to create mutually exclusive sub-categories, we registered *material support* as one category and *material support and dissolution of the conjugal couple* as the second sub-category.
The fulfillment of the basic needs (food, clothes, shoes, heating)	We distinguished between the cases in which the basic needs of the children (food, clothes, shoes, heating) *were fulfilled* and the cases in which the basic needs of the children *were not* fulfilled.
The migrated parent	The following three types of cases emerged: the migrated parent was *the mother*, *the father*, or *both parents migrated*.
The guardian/child carer	The following two types of cases emerged: the cases in which the child carer was one of the parents and the case in which the child carers were the grandparents.
